# Not all Forms of Muscle Hypertonia Worsen With Fatigue: A Pilot Study in Para Swimmers

**DOI:** 10.3389/fphys.2022.902663

**Published:** 2022-06-22

**Authors:** Luca Puce, Nicola Luigi Bragazzi, Antonio Currà, Lucio Marinelli, Laura Mori, Filippo Cotellessa, Karim Chamari, Marta Ponzano, Mohammad Hossein Samanipour, Pantelis T. Nikolaidis, Carlo Biz, Pietro Ruggieri, Carlo Trompetto

**Affiliations:** ^1^ Department of Neuroscience, Rehabilitation, Ophthalmology, Genetics, Maternal and Child Health (DINOGMI), University of Genoa, Genoa, Italy; ^2^ Laboratory for Industrial and Applied Mathematics (LIAM), Department of Mathematics and Statistics, York University, Toronto, ON, Canada; ^3^ Department of Medical-Surgical Sciences and Biotechnologies, Academic Neurology Unit, Ospedale A. Fiorini, Terracina, Sapienza University of Rome, Polo Pontino, Italy; ^4^ Istituto di Ricovero e Cura a Carattere Scientifico (IRCCS) Ospedale Policlinico San Martino, Genoa, Italy; ^5^ Aspetar, Orthopaedic and Sports Medicine Hospital, FIFA Medical Centre of Excellence, Doha, Qatar; ^6^ ISSEP Ksar-Said, La Manouba University, Manouba, Tunisia; ^7^ Department of Health Sciences (DISSAL), Section of Biostatistics, University of Genoa, Genoa, Italy; ^8^ Department of Sport Science, Imam Khomeini International University, Qazvin, Iran; ^9^ School of Health and Caring Sciences, University of West Attica, Athens, Greece; ^10^ Orthopedics and Orthopedic Oncology, Department of Surgery, Oncology and Gastroenterology (DiSCOG), University of Padova, Padova, Italy

**Keywords:** stretch reflex, spontaneous tonic muscle excitation, pain, median frequency, paralympic sport classes

## Abstract

In hypertonic muscles of patients with upper motor neuron syndrome (UMNS), investigation with surface electromyography (EMG) with the muscle in a shortened position and during passive muscle stretch allows to identify two patterns underlying hypertonia: spasticity and spastic dystonia. We recently observed in Para swimmers that the effect of fatigue on hypertonia can be different from subject to subject. Our goal was, therefore, to understand whether this divergent behavior may depend on the specific EMG pattern underlying hypertonia. We investigated eight UMNS Para swimmers (five men, mean age 23.25 ± 3.28 years), affected by cerebral palsy, who presented muscle hypertonia of knee flexors and extensors. Muscle tone was rated using the Modified Ashworth Scale (MAS). EMG patterns were investigated in *rectus femoris* (RF) and *biceps femoris* (BF) before and after two fatiguing motor tasks of increasing intensity. Before the fatiguing tasks, two subjects (#2 and 7) had spasticity and one subject (#5) had spastic dystonia in both RF and BF. Two subjects (#3 and 4) showed spasticity in RF and spastic dystonia in BF, whereas one subject (#1) had spasticity in RF and no EMG activity in BF. The remaining two subjects (#6 and 8) had spastic dystonia in RF and no EMG activity in BF. In all the 16 examined muscles, these EMG patterns persisted after the fatiguing tasks. Spastic dystonia increased (*p* < 0.05), while spasticity did not change (*p* > 0.05). MAS scores increased only in the muscles affected by spastic dystonia. Among the phenomena possibly underlying hypertonia, only spastic dystonia is fatigue-dependent. Technical staff and medical classifiers should be aware of this specificity, because, in athletes with spastic dystonia, intense and prolonged motor activity could negatively affect competitive performance, creating a situation of unfairness among Para athletes belonging to the same sports class.

## Introduction

To balance inclusion and fairness in Paralympic competitions, swimmers with an eligible impairment are categorized into sport classes based on their level of function (Burkett et al., 2018). According to “World Para Swimming Classification Rules and Regulations” document (World Para Swimming, 2018), each physically impaired swimmer is required to attend a medical and technical assessment, to evaluate the extent to which their impairment limits their swimming performance. They are then assigned to a sport class ranging from 1 (most severe activity limitation) to 10 (least activity limitation) (Payton et al., 2020).

Muscle tone increase (i.e., muscle hypertonia) is one of the eligible physical impairments in Paralympic sports (Hogarth et al., 2019). It is defined as the increase of resistance perceived by the clinician when moving a joint through a range of movement while the subject tries to keep muscles fully relaxed. Muscle hypertonia can hinder function and may even result in pain and complications (Ganguly et al., 2021). It is present in about 40% of Para swimmers belonging to low-medium sport classes (Puce et al., 2020).

Most Para athletes with muscle hypertonia are affected by the Upper Motor Neuron Syndrome (UMNS), i.e., a constellation of signs and symptoms due to the disruption of the central motor command to the spinal motor nuclei. Muscle weakness is considered a negative sign, since it is due to a reduction of muscle excitation (i.e., diminished neural input to the muscle). Other UMNS signs are characterized by an exaggerated muscle excitation. These are called positive signs and include spasticity, spastic dystonia, muscle spasms and co-contraction. Cerebral palsy (CP) is one of the diseases that can lead to UMNS; the others include stroke, multiple sclerosis, and traumatic lesions of the central nervous system (Trompetto et al., 2014).

In UMNS subjects, muscle hypertonia can be due to spasticity or spastic dystonia (Trompetto et al., 2019a) (Puce et al., 2021).

Spasticity is an exaggerated stretch reflex (Thilmann et al., 1991). In normotonic subjects, passive movements performed during tone assessment are too slow to activate the stretch reflex. In these subjects, to evoke a stretch reflex, passive muscle stretch must be very fast, as the one produced by the tendon hammer. On the contrary, in subjects with spasticity, stretch reflex excitability is increased; consequently, stretch reflex can be activated also during tone assessment and this activation increases with fast stretches, leading to velocity-dependent hypertonia (Thilmann et al., 1991). Muscles are fully relaxed only when they are not subjected to passive stretching and when patients succeed in voluntarily suppressing excitation (Burke, 1988).

On the contrary, spastic dystonia is the inability to voluntarily silence muscle activity (Trompetto et al., 2019b). As a consequence, it is characterized by a spontaneous tonic muscle activation while the subject tries to relax (Gracies, 2005). Although spastic dystonia is present without any muscle stretch, it is stretch-sensitive, meaning that it increases when the muscle is stretched, leading to velocity-dependent hypertonia, as occurring in spasticity (Marinelli et al., 2017). Table 1 shows the clinical and EMG features of spasticity and spastic dystonia.

**TABLE 1:** Difference between spasticity, and spastic dystonia. Subject is asked to stay completely relaxed during assessment.

	Clinical features		EMG features		
	Pathological posture at rest	Passive movement	Muscle held in a short position	Muscle elongation	Muscle held in a stretched position
Spasticity	None	Velocity-dependent hypertonia	No activity	Velocity-dependent EMG activity	No activity
Spastic dystonia	Present	Velocity and length-dependent hypertonia	Tonic activity	Velocity-dependent EMG activity	Tonic activity

With clinical examination alone, spasticity cannot be distinguished from spastic dystonia (Trompetto et al., 2020). Therefore, electromyographic (EMG) evaluation is indispensable (Marinelli et al., 2017) (Trompetto et al., 2019a) (Puce et al., 2021).

We have preliminarily reported that CP Para athletes with hypertonia belonging to the same sport class (i.e., having comparable functional condition) may respond to fatigue differently. Whereas in some athletes hypertonia does not change with fatigue, in others it becomes increasingly intense and painful (Puce et al., 2018).

We hypothesize that the distinct influence of fatigue originates from the underlying mechanism producing hypertonia (i.e., spasticity or spastic dystonia). In this case, some Para swimmers may have an advantage over others, and it would be necessary, in order to ensure fairness in competitions, to distinguish athletes with spasticity from those with spastic dystonia.

Previous studies show that fatigue modifies both cortico-spinal (Kuppuswamy et al., 2015) and stretch reflex excitability (Biro and Gri, 2007), likely impacting both spasticity and spastic dystonia.

The present study investigates a group of CP Para swimmers affected by hypertonia in knee flexors and extensors. The two aims of the study are: 1) to identify the muscles affected by spasticity or spastic dystonia by means of surface EMG (Puce et al., 2021); and, 2) to investigate the effects of fatigue on spasticity and spastic dystonia.

## Methods

### Participants

Para swimmers were selected based on the following inclusion criteria:

• CP diagnosis
• Cognitive functioning sufficient to give informed consent and to understand simple instructions (e.g., to remain relaxed during evaluation)
• Modified Ashworth Scale (MAS) score >0 in both knee extensors and flexors
• Normal passive range of motion (p-ROM) of knee joint (i.e., from the leg fully extended to the calf pushing onto the back of the thigh)
• No pain during knee joint passive mobilization

Exclusion criteria were: 1) Athletes with any additional pathological conditions that may affect motor function, and 2) the use of intrathecal baclofen and botulinum toxin treatment up to 8 months prior to the enrollment.

The study was carried out in accordance with the code of ethics of the World Medical Association for experiments involving humans (Declaration of Helsinki 2014). A written informed consent was obtained from all participants prior to enrollment. The project was approved by the local Ethical Committee (University of Genova, Italy. N. 2020/21).

### Clinical Assessment

Gross motor function was classified using the expanded and revised version of the Gross Motor Function Classification System (GMFCS) (Palisano et al., 2008). Tone of knee flexors and extensors was bilaterally rated according to the MAS (Bohannon and Smith, 1987). Pain during passive mobilization of knee joint was rated using a Numeric Rating Scale (pain NRS), with 0 being “no pain” and 10 being “the worst pain imaginable” (Price et al., 1994).

### EMG and Kinematic Recordings

EMG signals from *rectus femoris* (RF) and *biceps femoris* (BF) were recorded through bipolar surface electrodes positioned according to SENIAM guidelines (Hermens et al., 2000) from the most hypertonic side.

The EMG signals were acquired using wireless EMG equipment (Cometa Srl, Milan, Italy) with a band-pass filter of 1st order in the range of 10–500 Hz and digitized at 2000 samples/s. Raw EMG signals were processed with a band-pass Butterworth filter of 4th order bidirectionally in the range of 20–500 Hz and full wave rectified With this technique, the transfer function of the filter is real, and, therefore, no delay or phase distortion is applied to the signal to be filtered. During passive movements, knee joint angle was continuously acquired using a twin-axis electronic goniometer (TSD130b, Biopac System Inc., United States).

### EMG Assessment of Spontaneous Tonic Muscle Excitation and Stretch Reflex

**FIGURE 1:**
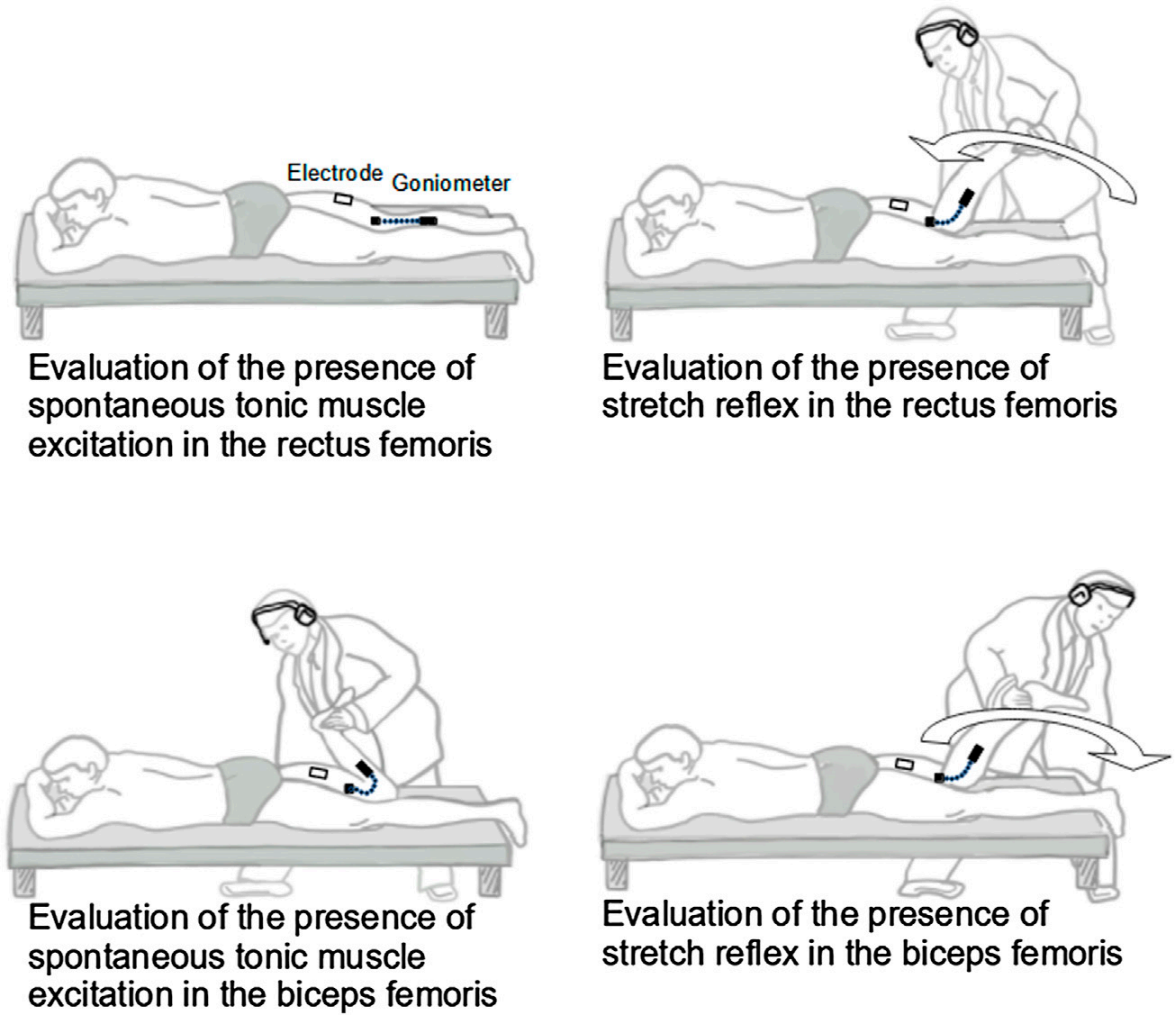
Experimental set-up and procedure.

Subjects were lying on a stretcher in the prone position and asked to remain completely relaxed and silent (Figure 1).

To seek spontaneous tonic muscle excitation, the examined muscle (RF or BF) was held in the short position (i.e., flexed knee for BF, and extended knee for RF). After a relaxation period lasting 1 min, during which the subject was invited to remain completely relaxed, spontaneous tonic muscle excitation was assessed by recording the EMG for 30 s. If a tonic EMG activity was detected for at least 20 s, spontaneous tonic muscle excitation was considered present, and it was measured by calculating the Average Rectified Value (ARV [µV]) of the entire 30-second period to allow the comparison of activations of different duration. EMG recording was visually inspected, and EMG activity was considered present if signals resembling motor unit potentials’ shape were clearly distinguishable from background noise.

To evaluate the stretch reflex in RF, immediately after completing the 30-second EMG recording aimed at investigating spontaneous tonic muscle excitation, the examiner moved the subject’s leg to maximum flexion in one second. To evaluate the stretch reflex in BF, the examiner moved the subject’s leg to maximum extension in one second. A method developed in our laboratory was used to control the duration of passive displacement. The method is based on acoustic feedback produced by an electronic metronome set at 60 beats per minute and delivered to the examiner *via* headphones. Therefore, the interval between two consecutive tones corresponded to one second. First, the examiner applied continuous manual knee displacements at a constant pace, so that the leg arrived at the extreme flexed and extended positions in synchrony with consecutive tones (duration of passive movement of one second). Second, when the examiner had synchronized the movement with the tones, the movement was stopped at one extreme position (leg flexed or extended). This position was maintained for 90 s (60 s of relaxation period and 30 s to assess spontaneous tonic muscle excitation, as described above). During these 90 s, the examiner continued to conceptualize the previously executed movement using first perspective motor imagery (Schuster et al., 2011) with the metronome tone as a cue. Third, after the 90 s of motor imagery, the examiner moved the leg so that it arrived at the opposite position in synchrony with the following tone (Marinelli et al., 2013).

Stretch reflex was assessed by recording the EMG during the one-second leg displacement. If EMG activity in this period clearly stood up from that recorded in the preceding EMG (i.e., 30 s EMG used to assess spontaneous tonic muscle excitation), stretch reflex was considered present, and it was measured by calculating the ARV of the entire one-second period.

When the stretch reflex—but not spontaneous tonic muscle excitation—was present, the hypertonic muscle was considered affected by spasticity. When both spontaneous tonic muscle excitation and stretch reflex were present, the hypertonic muscle was considered affected by spastic dystonia (Puce et al., 2021).

### Fatigue Test and EMG Assessment

Cybex II isokinetic dynamometer (Cybex, Division of Lumex Inc. Ronkonkoma, NY) was used for the fatigue test. The Para swimmer was sitting and harnessed to a Cybex chair with the hip flexed at 90°, with the rotational axis of the dynamometer aligned to the lateral femoral condyle. Each athlete underwent two fatigue tests: *test-1* and *test-2*, both administered at ≃10 a.m. 1 week apart. Clinical and EMG assessment of both spontaneous tonic muscle excitation and stretch reflex were performed just before (T_0_) and just after (T_1_) *test-1*, and just after *test-2* (T_2_). After an individual standardized warm-up, the subject was asked to perform continuous maximal concentric flexion-extension knee movements at 60°/s on the isokinetic dynamometer. A ROM of 85° was set (from 90° of knee flexion to 175° of knee extension). Subjects received verbal encouragement and visual torque feedback during the tests. Muscle fatigue *test-1* consisted in 15 knee flexion-extension movements, while muscle fatigue *test-2* consisted in 35 knee flexion-extension movements.

This method was chosen to induce in the participants two different metabolic loads.

Changes in motion duration and ROM across the cycles were not allowed, therefore, it can be expected that *test-1* is less demanding than *test-2* in terms of biochemical processes attributable to fatigue (Faude et al., 2009).

**FIGURE 2:**
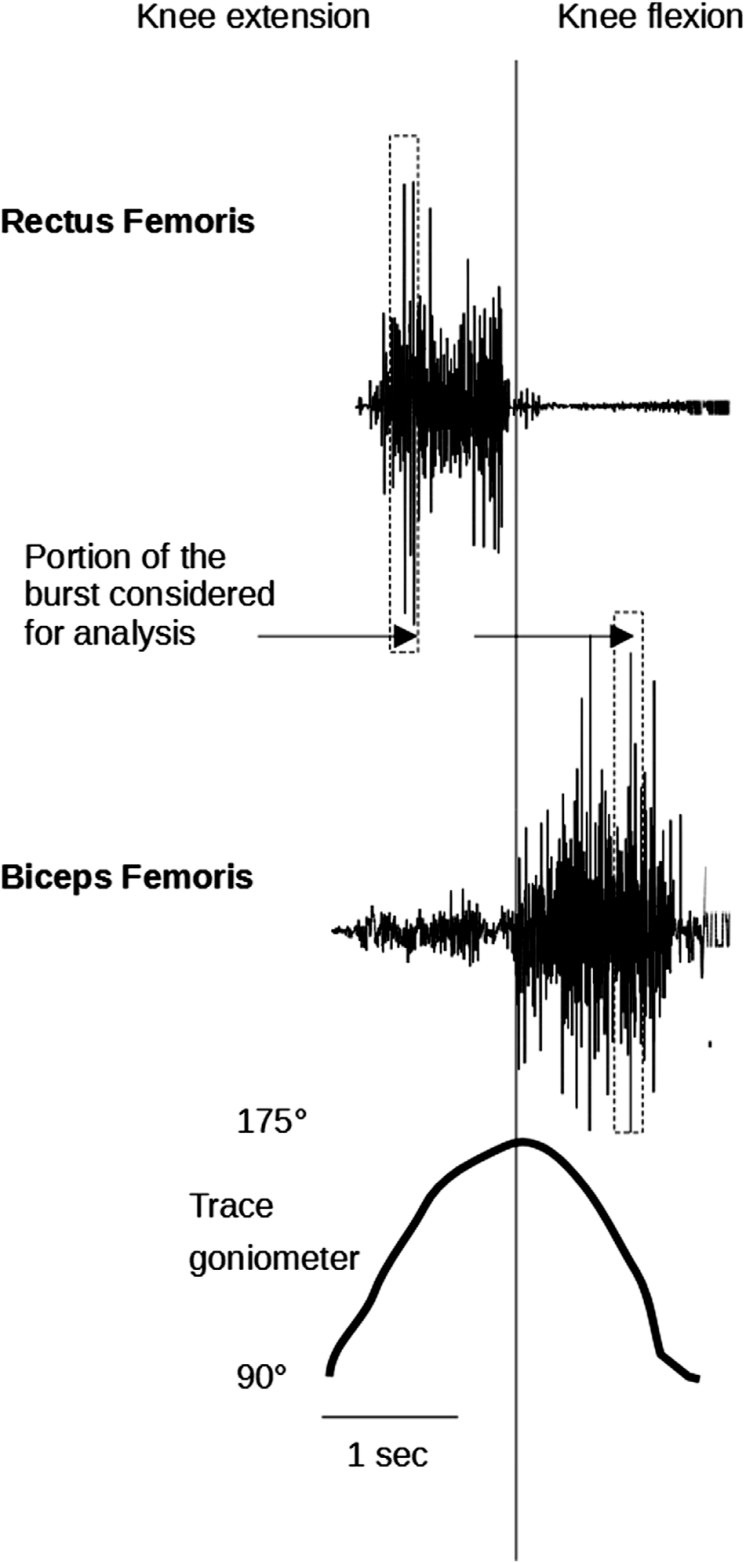
The figure depicts a raw surface myoelectric signal recorded during two repetitions of extension/flexion of the knee during the fatigue test with isokinetic device. The vertical rectangular bands identify the portion of the burst considered for analysis of EMG spectrum in each cycle.

The median frequency of the EMG power spectrum (MDF [Hz]) was used to evaluate muscle fatigue in RF and BF during *tests-1* and *2*. The EMG spectrum was estimated in a window of 480 samples corresponding to the ROM between 125° and 145° for knee extensions and between 145° and 125° for knee flexions (Figure 2). The Fast Fourier Transform (FFT) function was used to perform a linear magnitude FFT on the movement of the selected data portions. MDF was then defined as the harmonic corresponding to the 50th percentile of the energy distribution in the frequency spectrum. Open-source Python software distributed by Anaconda Inc. was used to calculate these parameters.

Performing these operations gave a plot of MDF values versus time. To estimate the time evolution of MDF, a linear fitting of the data set was performed, and the slope (time-slope of MDF) was extracted. The slopes were finally normalized to the value of the regression line at the initial time of the first analyzed excitation interval. Slopes were expressed as percentage values. A progressive decrease in MDF throughout the exercise is indicative of muscle fatigue.

### Statistical Analysis

Continuous variables were computed as means and standard deviations, whilst categorical parameters were expressed as percentages, where appropriate. Normality of data distribution was checked using the Shapiro-Wilk’s test, which was preferred over other normality tests given the small sample size utilized in the present investigation.

Besides descriptive statistics, repeated measurements analysis was conducted at different time points for the following three groups: “no EMG activity”, “spasticity” and “spastic dystonia”. For time trend analysis, both linear and quadratic trends were assessed. Moreover, Mauchly’s sphericity test was conducted to verify the assumptions underlying repeated measures. In case of violation of sphericity, if epsilon was less than 0.75, the Greenhouse-Geisser’s correction was applied, otherwise, the Huynh-Feldt’s correction was applied. Pairwise comparisons were carried out, computing mean differences, with their 95% confidence interval (95%CI) and standard error (SE), adjusting for multiple comparisons according to Bonferroni.

All statistical analyses were conducted using the commercial software “Statistical Package for the Social Sciences” (SPSS version 28, IBM Corp., Armonk, NY, United States). For all analyses, *p*-value <0.05 was considered statistically significant. Graphs were generated using the commercial software MedCalc (MedCalc Statistical Software version 20.011, MedCalc Software Ltd., Ostend, Belgium).

## Results

According to the inclusion criteria, eight CP Para swimmers (5 men, 62.5%, 3 women, 37.5%; mean age 23.25 ± 3.28 years, median 23 years) were enrolled.

Their demographic, disease-related, and athletic characteristics are reported in Table 2.

**TABLE 2:** Demographic, disease-related, and athletic characteristics of the 8 subjects included in the study. The prefix “S” corresponds to freestyle. There are ten sport classes for this style (1-10). Para swimmers with greater physical limitations compete in lower classes. Abbreviations: GMFCS, Gross Motor Function Classification System; CP, cerebral palsy; H, hemiplegic; P, paraplegic; T, tetraplegic.

Para athlete		1	2	3	4	5	6	7	8
Sex		M	F	M	F	M	M	F	M
Age (years)		22	24	27	25	28	20	19	21
Sport class		S4	S5	S6	S6	S5	S6	S4	S5
GMFCS		III	II	II	II	II	II	III	III
CP topography		T	H	H	H	T	P	T	T
Training (min/week)	In water	660	840	960	600	900	540	360	720
	Dry land	0	30	90	90	90	30	0	30
	Stretching	0	10	30	30	30	10	30	10
International competitive experience (years)		6	7	7	8	14	11	2	4

### EMG Findings

Two subjects (#2 and 7) had spasticity and one subject (#5) had spastic dystonia in both RF and BF. Two subjects (#3 and 4) showed spasticity in RF and spastic dystonia in BF, whereas one subject (#1) had spasticity in RF and no EMG activity in BF. The remaining two subjects (#6 and 8) had spastic dystonia in RF and no EMG activity in BF.

**FIGURE 3:**
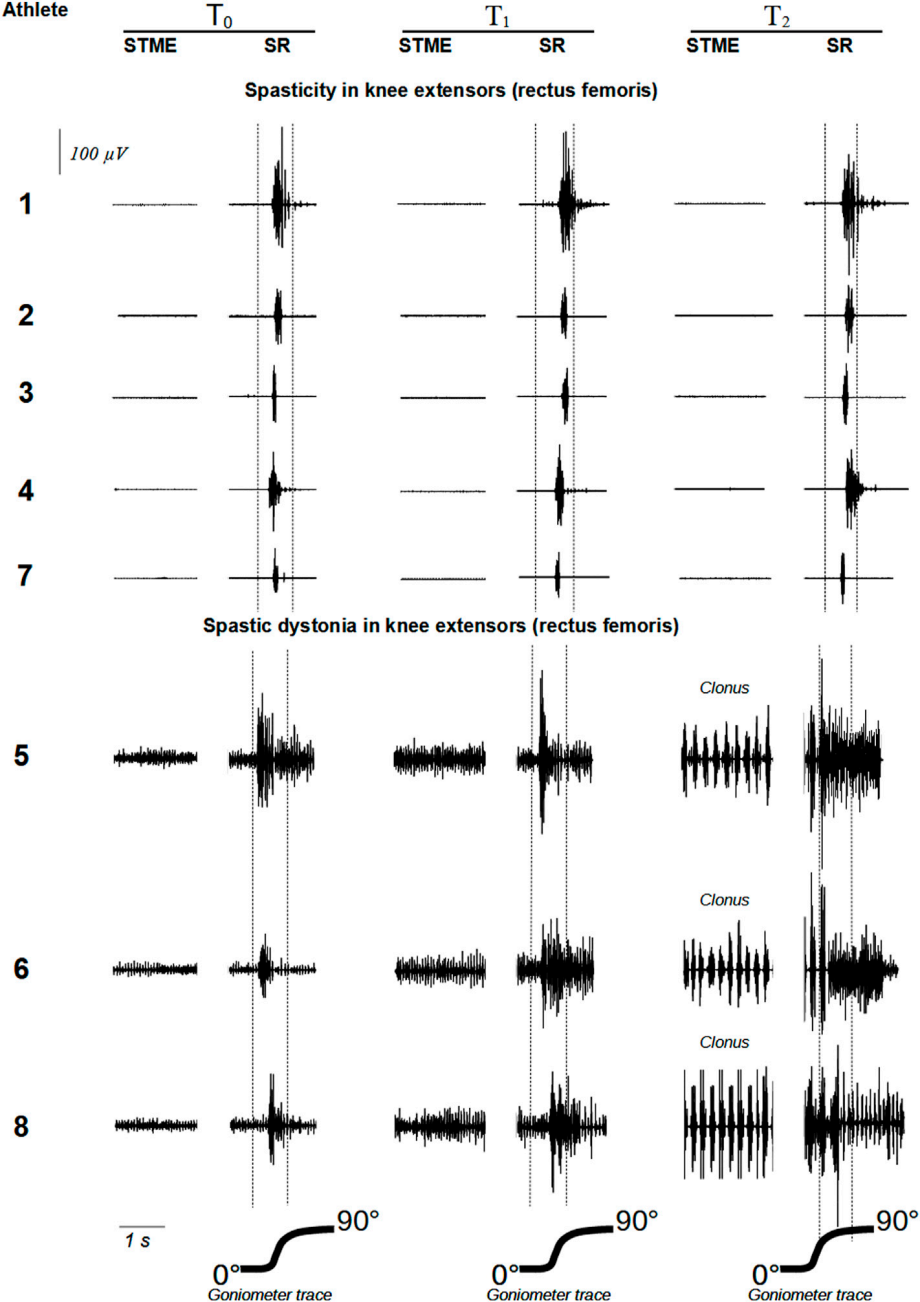
show raw EMG signals from *rectus femoris* and *biceps femoris*, respectively. Abbreviations: STME (spontaneous tonic muscle excitation); SR (stretch reflex).

**FIGURE 4:**
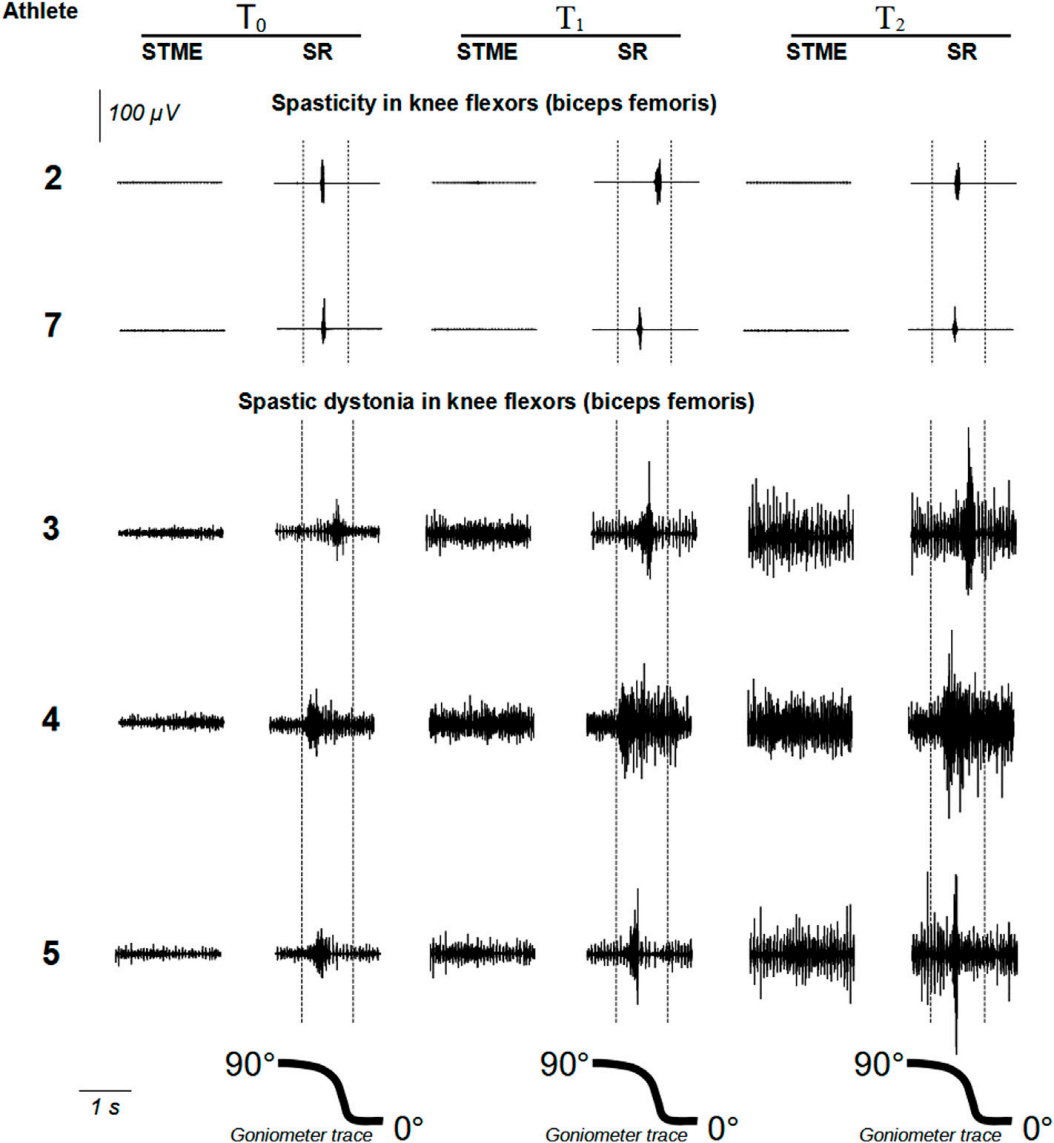
show raw EMG signals from *rectus femoris* and *biceps femoris*, respectively. Abbreviations: STME (spontaneous tonic muscle excitation); SR (stretch reflex).

For each one of the 16 muscles examined (eight from the left and eight from the right side), the EMG pattern detected at T_0_ (i.e., spasticity or spastic dystonia) remained in the subsequent recording sessions (T_1_ and T_2_) (Figures 3, 4).

### Rectus Femoris: Fatigue and EMG Patterns

In the five muscles with spasticity, fatigue differed between *test-1* and *test-2*. Mean difference was -12.66% ± 3.68 [(95%CI −17.23 to −8.09), t = −7.69, *p* = 0.0015]. Similarly, in the three muscles with spastic dystonia, fatigue differed between *test-1* and *test-2*. The mean difference was −8.50% ± 2.46 [(95%CI −14.61 to −2.39), t = −5.99, *p* = 0.0268] (Table 2).

In the five muscles affected by spasticity, stretch reflex showed no significant trend among time points (F = 1.09, *p* = 0.359) (Table 3; Figure 3).

**TABLE 3:** Average rectified value (ARV) of spontaneous tonic muscle excitation (STME) and stretch reflex along the three time points (T_0_, T_1_ and T_2_). Slope values of median frequency (MDF) regression line along the 15 (*test-1*) and 35 (*test-2*) flexion movements.

Athlete	T_0_ [ARV (μV)]		*Test-1* slope MDF [% (Hz)]	T_1_ (ARV μV)		*Test-2* slope MDF [% (Hz)]	T_2_ [ARV (μV)]	
	STME	SR		STME	SR		STME	SR
Spasticity rectus femoris								
1		26.9	−15.1		27.6	−22.6		26.6
2		12.3	−7.0		11.2	−24.7		14.9
3		12.3	−4.0		12.4	−17.9		13.5
4		17.7	−7.0		17.6	−19.3		17.7
7		8.9	−8.4		9.9	−20.3		9.5
MEAN		15.6	−8.7		15.8	−21.7		16.4
SD		7.1	5.7		7.2	3.5		6.4
Spastic dystonia rectus femoris								
5	6.0	22.7	−3.8	9.0	33.3	−9.5	22.6	46.1
6	3.7	11.5	−8.1	6.8	22.5	−18.4	19.7	35.4
8	3.6	20.4	−5.2	8.0	25.4	−14.7	24.4	35.2
MEAN	4.4	18.2	−5.7	7.9	27.1	−14.2	22.2	38.9
SD	1.4	5.9	2.2	1.1	5.6	4.5	2.4	6.2
No EMG activity biceps femoris								
1			−3.8			−6.4		
6			−3.3			−8.5		
8			−1.8			−6.8		
MEAN			−3.0			−7.2		
SD			1.0			1.1		
Spasticity biceps femoris								
2		7.4	−3.2		7.5	−15.6		6.2
7		7.6	−11.9		7.4	−18.4		8.2
MEAN		7.5	−7.6		7.4	−17.0		7.2
SD		0.1	6.2		0.1	2.0		1.4
Spastic dystonia biceps femoris								
3	3.7	12.2	−8.3	9.7	25.5	−16.1	19.9	33.4
4	4.5	10.3	−5.0	9.5	22.5	−10.7	15.5	30.5
5	3.4	9.6	−3.6	7.2	30.5	−10.6	19.6	37.9
MEAN	3.9	10.7	−5.6	8.8	26.1	−12.5	18.3	33.9
SD	0.6	1.3	2.4	1.4	4.1	3.1	2.5	3.8

On the contrary, in the three muscles affected by spastic dystonia, spontaneous tonic muscle excitation showed an increasing variation (t = 11.79, *p* = 0.0071 for linear increase; t = 16.80, *p* = 0.0035 for quadratic increase), and differed at each time point (F = 147.13, *p* = 0.007). More specifically, T_0_ differed from T_1_ [mean difference −3.50 (95%CI −6.95 to −0.05), SE = 0.45, *p* = 0.0486] and T_2_ [mean difference −17.80 (95%CI −29.35 to −6.25), SE = 1.51, *p* = 0.0214]. T_1_ differed from T_2_ [mean difference −14.30 (95%CI −22.48 to −6.12), SE = 1.07, *p* = 0.0166]. Stretch reflex also showed an increasing variation (F = 47.90, *p* = 0.020), which was more likely to be linear (t = 7.01, *p* = 0.0198) rather than quadratic (t = 3.22, *p* = 0.0843). It did not significantly change at the different time points, if not between T_1_ and T_2_ [mean difference −11.83 (95%CI −19.61 to −4.05), *p* = 0.0219] (Table 3; Figure 3).

### Biceps Femoris: Fatigue and EMG Patterns

In the three muscles with no EMG activity, and in the two muscles affected by spasticity, fatigue did not differ between *test-1* and *test-2*. Mean difference was −9.45% ± 4.17 [(95%CI −46.93 to 28.03), t = −3.203, *p* = 0.1926] and −4.27 ± 1.45 [(95%CI: −7.86; −0.67), t = −5.1078, *p* = 0.0363] respectively.

In the three muscles affected by spastic dystonia, fatigue differed significantly between *test-1* and *test-2*. The mean difference was -6.83% ± 1.06 [(95%CI −9.47 to −4.20), t = −11.17, *p* = 0.0079] (Table 3).

In the two muscles with spasticity, stretch reflex show no significant trend among endpoints (F = 0.080, *p* = 0.824) (Table 3; Figure 4).

On the contrary, in the three muscles affected by spastic dystonia, spontaneous tonic muscle excitation exhibited an increasing variation (which was more likely to be linear, t = 8.35, *p* = 0.0141, rather than quadratic, t = 2.09, *p* = 0.1720). It differed at various time points (F = 46.80, *p* = 0.013). More specifically, T_0_ differed from T_1_ [mean difference −4.93 (95%CI −9.80 to −0.07), SE = 0.64, *p* = 0.0486] and T_2_ [mean difference −14.47 (95%CI −27.73 to −1.21), SE = 1.73, *p* = 0.0422]. T_1_ did not significantly differ from T_2_ [mean difference = −9.53 (95% CI −23.88 to 4.81), SE = 1.88, *p* = 0.1101]. Stretch reflex also showed an increasing variation (which was more likely to be linear, t = 9.11, *p* = 0.0118, rather than quadratic, t = −2.64, *p* = 0.1188). Variations were significant at various time points (F = 59.88, *p* = 0.016). T_0_ differed from T_2_ [mean difference −23.23 (95%CI −42.74 to −3.73), SE = 2.55, *p* = 0.0355]. T_1_ differed from T_2_ [mean difference −7.77 (95%CI −9.19 to −6.35), SE = 0.19, *p* = 0.0017]. However, T_1_ did not significantly differ from T_0_ [mean difference = −15.47 (95%CI = −36.37; 5.43), SE = 2.74, *p* = 0.0897] (Table 3; Figure 4).

### Clinical Findings

In the six muscles affected by spastic dystonia, MAS, and pain NRS scores obtained at baseline (T_0_), after *test-1* (T_1_) and after *test-2* (T_2_) progressively increased (Table 4). In the remaining ten muscles affected by spasticity or showing no EMG activity, the MAS scores remained unchanged (80% muscles) or decreased (20% muscles); whereas the pain NRS score remained unchanged (70% muscles) or increased (30% muscles) (Table 4).

**TABLE 4:** Values in the Modified Ashworth Scale (MAS) and Numeric Rating Scale for the pain (NRS) along the three time points (T_0_, T_1_ and T_2_).

Athlete	T_0_		T_1_		T_2_	
	MAS	NRS	MAS	NRS	MAS	NRS
Spasticity knee extensor (rectus femoris)						
1	1.5	0.0	1.5	0.0	1.5	2.0
2	1.5	0.0	1.5	0.0	1.5	0.0
3	1.0	0.0	1.0	0.0	1.0	2.0
4	1.5	0.0	1.0	3.0	1.0	2.0
7	1.0	0.0	1.0	0.0	1.0	0.0
MEAN	1.3	0.0	1.2	0.6	1.2	1.2
SD	0.3	0.0	0.3	1.3	0.3	1.1
MEDIAN	1.5	0.0	1.0	0.0	1.0	2
Spastic dystonia knee extensor (rectus femoris)						
5	1.5	0.0	2.0	3.0	4.0	8.0
6	2.0	0.0	3.0	3.0	4.0	7.0
8	1.0	0.0	2.0	2.0	4.0	6.0
MEAN	1.5	0.0	2.3	2.7	4.0	7.0
SD	0.5	0.0	0.6	0.6	0.0	1.0
MEDIAN	1.5	0.0	2.0	3.0	4.0	7.0
No EMG activity knee flexor (biceps femoris)						
1	1.0	0.0	1.0	0.0	1.0	0.0
6	1.0	0.0	1.0	0.0	1.0	0.0
8	1.0	0.0	1.0	0.0	1.0	0.0
MEAN	1.0	0.0	1.0	0.0	1.0	0.0
SD	0.0	0.0	0.0	0.0	0.0	0.0
MEDIAN	1.0	0.0	1.0	0.0	1.0	0.0
Spasticity knee flexor (biceps femoris)						
7	1.5	0.0	1.5	0.0	1.5	0.0
2	1.5	0.0	1.0	0.0	1.0	0.0
MEAN	1.5	0.0	1.3	0.0	1.3	0.0
SD	0.0	0.0	0.4	0.0	0.4	0.0
MEDIAN	1.5	0.0	1.3	0.0	1.3	0.0
Spastic dystonia knee flexor (biceps femoris)						
3	1.0	0.0	2.0	3.0	3.0	5.0
4	2.0	0.0	3.0	4.0	3.0	6.0
5	2.0	0.0	3.0	3.0	3.0	5.0
MEAN	1.7	0.0	2.7	3.3	3.0	5.3
SD	0.6	0.0	0.6	0.6	0.0	0.6
MEDIAN	2.0	0.0	3.0	3.0	3.0	5.0

## Discussion

In the muscles affected by spastic dystonia (both RF and BF), there was an increasing trend both for MAS and pain NRS at the different time points. On the contrary, in the muscles showing no EMG activity or in those affected by spasticity, MAS and pain NRS values did not vary among time points (Table 4).

These data confirm our preliminary clinical observations, i.e., that CP Para swimmers of the same sport class respond differently to fatigue. Whereas some experience no aggravation of muscle hypertonia, others manifest increasingly intense and painful hypertonia (Puce et al., 2018).

### EMG Findings at Baseline (T_0_)

All the eight RF muscles exhibited a stretch reflex that vanished as soon as the passive movement was stopped or just after a few seconds. Three athletes had spontaneous tonic EMG excitation indicating spastic dystonia, five had not, indicating spasticity. Of the eight BF muscles, three exhibited no EMG activity at rest nor during passive movement.

Reasonably, in these subjects the synergistic muscles (i.e., semimembranosus muscle and semitendinosus muscle) are responsible for knee flexor hypertonia.

However, it cannot be ruled out that these were normal muscles with overestimated tone scores, as they had a lower MAS score than the others (Table 4). The remaining five muscles exhibited a stretch reflex (three spastic dystonia, and two spasticity). Therefore, in Para swimmers the EMG patterns in knee flexors and extensors replicates those observed in multiple sclerosis patients, i.e., spastic dystonia is evenly distributed between flexor and extensor muscles, whereas spasticity prevails in extensors (Marinelli et al., 2017; Puce et al., 2021).

### EMG Findings and Fatigue

In all 16 muscles, the EMG pattern found at T_0_ (i.e., spasticity or spastic dystonia) remained unchanged at T_1_ and T_2_. This observation suggests that each patient expresses a predominant EMG pattern over time.

Both *test-1* and *test-2* were effective in causing fatigue because regression analysis showed that the EMG signal decreased both at T_1_ and T_2_ in all the sixteen muscles examined.

After intense physical exercise with muscle fatigue, the 6 muscles affected by spastic dystonia undergo an increase in spastic dystonia. On the other hand, in the seven muscles affected by spasticity and in the three muscles without EMG activity, physical exercise does not cause any modification of the EMG picture.

These consistent data demonstrate for the first time that, in the RF muscle and in the BF muscle, spastic dystonia increases with muscle fatigue, while spasticity remains unchanged. This is the main result of our work.

In muscles affected by spastic dystonia, the increase in tone after muscle fatigue is justified by the increase in spastic dystonia in the investigated muscle (RF or BF), regardless of the behavior of the synergistic muscles.

In muscles affected by spasticity or in muscles without any involuntary EMG activity, the non-variation in tone during muscle fatigue suggests that, even in the synergistic muscles, as in the muscles evaluated (RF or BF), fatigue does not induce any EMG increase during muscle stretching, otherwise muscle tone would have increased.

Increased spontaneous tonic muscle excitation after intense and repeated voluntary muscle contractions is expected in spastic dystonia, because spastic dystonia causes inability to voluntarily relax muscles once contracted. Increased stretch reflex likely depends on the increased spontaneous tonic muscle excitation, that makes spinal motor neurons more intensely and more easily excitable by sensory inputs.

Reasonably, during passive muscle stretching, increasing hypertonia causes pain. Also in healthy muscles the eccentric contraction (that lengthens a contracting muscle) causes pain, by disrupting individual muscle fibers and releasing algogenic substances (Chang et al., 2013).

### Limitations of the Study

A limited sample of participants was investigated. Additionally, only one joint was assessed. All this limits the generalization of our results.

To evaluate the stretch reflex, a method similar to that used in clinical practice to assess muscle tone was used: rather slow manual passive mobilizations, covering the entire range of joint movement. Since the reflex is not only velocity-dependent but also length-dependent, with this method the latencies of the reflex are high, often higher than 300 ms, thus not allowing to exclude with certainty a voluntary component in the detected EMG activity.

Furthermore, since surface EMGs were collected with a single pair of electrodes positioned at a given point of interest in the skin, we cannot exclude that excitation of deep fibers in the target muscles was not properly sampled (Vigotsky et al., 2018) (Watanabe et al., 2021) (Vigotsky et al., 2022), leading to type II error (Vieira and Botter, 2021).

Despite the precautions taken, another limitation is the method of analysis of the MDF in the fatigue test. During the continuous maximum concentric flexion-extension movements of the knee, several factors related to the biomechanics of the task may have come into play, i.e., changes in muscle length (Vieira and Botter, 2021), in the forces exercised (Bilodeau et al., 1990) and in muscle architecture (Vieira et al., 2017). All these factors can contribute to the non-stationarity (rapid changes in signal properties) of the myoelectric signal (Bonato et al., 2001).

## Conclusion and Relevance of the Present Findings

Before fatigue, Para swimmers manifest similar hypertonia regardless of the underlying EMG pattern. After fatigue, only in subjects with spastic dystonia, hypertonia worsens, and pain develops. Technical staff and medical classifiers should be aware of this specificity, because in athletes with spastic dystonia, intense and prolonged motor activity could negatively impact competition performance, creating a situation of unfairness between Para athletes belonging to the same sports class. If the present preliminary results will be confirmed by larger studies, the swimming classification system should take into considerations that spastic dystonia worsens when fatigue develops, while spasticity and intrinsic hypertonia do not change.

## Data Availability

The original contributions presented in the study are included in the article/Supplementary Material, further inquiries can be directed to the corresponding author.
